# Improved cure rate of periprosthetic joint infection through targeted antibiotic therapy based on integrated pathogen diagnosis strategy

**DOI:** 10.3389/fcimb.2024.1388385

**Published:** 2024-05-21

**Authors:** Qijin Wang, Yongfa Chen, Yang Chen, Jianhua Lv, Haiqi Ding, Jiagu Huang, Jiexin Huang, Zida Huang, Bin Yang, Wenming Zhang, Xinyu Fang

**Affiliations:** ^1^ Department of Orthopaedic Surgery, The First Affiliated Hospital, Fujian Medical University, Fuzhou, China; ^2^ Department of Orthopaedic Surgery, National Regional Medical Center, Binhai Campus of the First Affiliated Hospital, Fujian Medical University, Fuzhou, China; ^3^ Fujian Provincial Institute of Orthopedics, The First Affiliated Hospital, Fujian Medical University, Fuzhou, China; ^4^ Department of Orthopaedic Surgery, The Affiliated Mindong Hospital of Fujian Medical University, Fuan, China; ^5^ Department of Laboratory Medicine, The First Affiliated Hospital, Fujian Medical University, Fuzhou, China

**Keywords:** periprosthetic joint infection, diagnosis, antibiotic, bacteria, total joint arthroplasty

## Abstract

**Objectives:**

This study aimed to determine whether combined of pathogen detection strategies, including specimen acquisition, culture conditions, and molecular diagnostics, can improve treatment outcomes in patients with periprosthetic joint infections (PJI).

**Methods:**

This retrospective study included suspected PJI cases from three sequential stages at our institution: Stage A (July 2012 to June 2015), Stage B (July 2015 to June 2018), and Stage C (July 2018 to June 2021). Cases were categorized into PJI and aseptic failure (AF) groups based on European Bone and Joint Infection Society (EBJIS) criteria. Utilization of pathogen diagnostic strategies, pathogen detection rates, targeted antibiotic prescription rates, and treatment outcomes were analyzed and compared across the three stages.

**Results:**

A total of 165 PJI cases and 38 AF cases were included in this study. With the progressive implementation of the three optimization approaches across stages A, B and C, pathogen detection rates exhibited a gradual increase (χ2 = 8.282, P=0.016). Similarly, utilization of targeted antibiotic therapy increased stepwise from 57.1% in Stage A, to 82.3% in Stage B, and to 84% in Stage C (χ2 = 9.515, P=0.009). The 2-year infection control rate exceeded 90% in both stages B and C, surpassing stage A (71.4%) (χ2 = 8.317, P=0.011). Combined application of all three optimized protocols yielded the highest sensitivity of 91.21% for pathogen detection, while retaining higher specificity of 92.11%.

**Conclusion:**

The utilization of combined pathogen diagnostic strategies in PJI can increase pathogen detection rates, improve targeted antibiotic prescription, reduce the occurrence of antibiotic complications, and achieve better treatment outcomes.

## Introduction

Despite significant advancements in implant sterilization techniques and aseptic surgical procedures in recent decades, periprosthetic joint infection (PJI) remains a major challenge for orthopedic surgeons (Ghimire and Song, 2021). The incidence of PJI persists at 1-3% after primary joint arthroplasty (Kapadia et al., 2016) and can be as high as 4-12% following revision surgery (Della Valle et al., 2011; Liu et al., 2014; Vrancianu et al., 2023). resulting in increased treatment costs and indirect elevation of patient mortality (Ghimire and Song, 2021).

Identification of causative pathogens is critical for effective management of infectious diseases (Kullar et al., 2022), including PJI (Parvizi et al., 2012, 2019). Microbiological culture plays a vital role in PJI diagnosis, involving preoperative aspiration of joint fluid for culture and intraoperative collection of multiple periprosthetic tissue specimens (Osmon et al., 2013; Stylianakis et al., 2018). However, conventional culture lacks perfect sensitivity and specificity, frequently resulting in missed diagnoses and treatment delays (Trampuz et al., 2007; Wouthuyzen-Bakker, 2023). Numerous factors collectively impact the accuracy of PJI diagnosis, such as inadequate preoperative joint fluid sampling, challenges in culturing intraoperative tissues, reduced planktonic bacteria in joint fluid due to biofilm formation (Jeyanathan et al., 2021), prior antibiotic exposure before culture (Malekzadeh et al., 2010), insufficient culture duration (Butler-Wu et al., 2011), improper specimen handling leading to microbial load loss (Van Cauter et al., 2018), and fastidious growth requirements of some pathogens (Brzezinski et al., 2021). These can contribute to culture-negative PJI (CN-PJI).

To address the aforementioned challenges in pathogen detection, targeted strategies must be implemented. Recent advances in specimen acquisition and processing have emerged, including ultrasound-guided aspiration, sonication of explanted prostheses (Trampuz et al., 2006; Rothenberg et al., 2017; Zhang et al., 2019), and tissue grinding (TF) (Fang et al., 2021b). Specialized culture techniques have been developed, such as prolonged incubation periods (Fang et al., 2021a), customized media (Fang et al., 2021a), and optimized temperature/humidity conditions. Furthermore, molecular assays like polymerase chain reaction (PCR) and metagenomic next-generation sequencing (mNGS) (Huang et al., 2020a; Mei et al., 2023) have been incorporated into diagnostic protocols for periprosthetic joint infection (PJI), resulting in improved pathogen detection rates.

In our institution, the stepwise implementation of the aforementioned optimization strategies led to observed improvements in pathogen detection rates and targeted antibiotic therapy efficacy. However, to our knowledge, there is currently no literature reporting on the combined pathogen diagnostic strategy for pathogen detection and guidance of antibiotic use in PJI. Given that our institution continuously improved and optimized pathogen diagnostic strategies to enhance the detection rate of pathogens causing PJI from 2012 to 2021, we intentionally segmented the entire study period into three phases: 2012 to 2015, 2015 to 2018, and 2018 to 2021.Therefore, the aim of this study is to evaluate the impact of gradually implemented optimized pathogen detection strategies on improving detection rates in PJI patients. We hypothesize that the stepwise incorporation of optimized techniques can significantly improve pathogen detection compared to conventional methods alone.

## Materials and methods

### Patient selection

This retrospective study was approved by the Institutional Review Board (2018 [026]) of our institution in accordance with the Declaration of Helsinki. Patients who underwent revision surgery for suspected PJI at our institution between July 2012 and June 2021 were enrolled. The study period was divided into three stages: Stage A (July 2012 to June 2015), Stage B (July 2015 to June 2018), and Stage C (July 2018 to June 2021). The inclusion criteria were: 1) patients with suspected PJI; 2) patients who underwent microbiological culture during diagnostic testing and treatment; and 3) patients with at least 2 years of follow-up after surgery. The exclusion criteria were: 1) patients with suspected pathogen contamination in culture results; 2) patients with concomitant infectious diseases other than PJI; and 3) patients with incomplete medical records. PJI diagnosis was based on the European Bone and Joint Infection Society (EBJIS) (McNally et al., 2021). Concurrently, patients with aseptic failure (AF) were enrolled as negative controls.

### Optimized specimen acquisition

#### Ultrasound-guided aspiration

Joint aspiration was carried out by surgeons (Authors 8, 10, and 11 the surgeons) under sterile conditions in the operating room. The puncture site was prepared with povidone iodine, and sterile needles were utilized for aseptic joint cavity aspiration without local anesthesia. Authors 8, 10, and 11 all received the same training on ultrasound-guided arthrocentesis and joint aspiration protocols and were experienced in performing ultrasound-guided joint aspirations. The preoperative aspiration protocol adhered to previously described guidelines (Della Valle et al., 2011). Ultrasound-guided aspiration enables real-time visualization of joint structure and needle trajectory, enhancing accuracy, safety and success rate of arthrocentesis compared to traditional landmark-guided aspiration. It allows precise needle placement into effusions while avoiding neurovascular structures.

#### Sonication of prostheses

The removed prostheses were placed in sterilized plastic containers containing approximately 400 ml of sterile saline solution. First, place in a vortex oscillator and oscillate for 30 seconds, followed by sonication using a 40 Hz ultrasound cleaner (VS-TP24 Ultrasonic Cleaner; Jiangsu Wuxi Woxin Instrument, Shanghai) for 5 minutes. The sonication fluid was then transferred into 50 ml centrifuge tubes and centrifuged at 4,000 rpm for 5 minutes (Eppendorf, 5430R, Germany). The supernatant was discarded, and the concentrated fluid was resuspended in sterile saline solution.

#### Tissue grinding

The periprosthetic tissue were collected from five different parts, cut into 0.5 cm^3^ pieces, placed into 3 ml of brain heart infusion (BHI) culture medium. After vortexing and shaking on a vortex oscillator for 15 minutes, the specimens were homogenized in an automated high-speed tissue homogenizer set at 40 Hz (JXFSTPRP-24; Jingxin Industrial, Shanghai, China) for 60 to 90 seconds.

### Conventional culture

A small volume (0.1ml) of synovial fluid and sonicate fluid from explanted prostheses was inoculated onto Columbia blood agar (Haibo Biotechnology, Qingdao, China) for culture under both aerobic and anaerobic conditions. The remaining specimen was directly injected into BACTEC Plus/F aerobic and anaerobic vials (Becton-Dickinson, Franklin Lakes, NJ, USA) followed by incubation in a BACTEC 9050 automated blood culture system (FX400; Becton-Dickinson) (Hughes et al., 2011). A small aliquot of tissue homogenate was inoculated onto Columbia blood agar (Haibo Biotechnology, Qingdao, China) for aerobic cultures.

### Optimized culture methods

#### Prolonged culture period

Based on preliminary clinical assessments, the incubation period was extended to 2-3 weeks for microorganisms with longer generation times (Ince et al., 2004; Schäfer et al., 2008).

#### Specialized culture conditions

Customized temperature, humidity, and atmosphere conditions (aerobic, CO2-enriched, microaerophilic, anaerobic) were implemented based on microbial growth requirements (Lewis et al., 2021). For example, Brucella agar plates were incubated in an anaerobic chamber with 5% CO2, 10% H2 and 85% N2 gas mixture for optimal recovery of anaerobes (Parvizi et al., 2016). Fungal cultures were incubated at 25-30°C in aerobic conditions. Fastidious bacteria were cultured in 5% CO2 at 35°C (Tande and Patel, 2014).

#### Specialized culture media

Targeted media were selected according to the specific nutritional needs of bacterial species (Lewis et al., 2021). For example, Sabouraud dextrose agar (Beiruite BioTech, Zhengzhou, China) was used for fungal detection. Selective enrichment agar (Thermo Fisher Scientific, USA) for Escherichia coli. Mannitol salt agar (Becton, Dickinson and Company, USA) for Staphylococcus aureus. Columbia Agar with 5% Sheep Blood (Becton, Dickinson and Company, USA) for Staphylococcus epidermidis. Blood agar (Thermo Fisher Scientific, USA) for Streptococcus species. Cetrimide agar (Becton, Dickinson and Company, USA) for Pseudomonas aeruginosa. BBE agar (Thermo Fisher Scientific, USA) for Enterococcus species. The reinforced clostridial medium (Thermo Fisher Scientific, USA) for Clostridium species. Additionally Anaerobic Agar (Thermo Fisher Scientific, USA) for Propionibacterium acnes.

### Molecular diagnostic methods

PCR amplification targeted the V4 region of the 16S rDNA using forward primer 515f (5’-GTGCCAGCMGCCGCGGTAA-3’) and reverse primer 806r (5’-GGACTACHVGGGTWTCTAAT-3’) for analysis of synovial fluid, sonicate prosthetic fluid and tissue homogenate specimens, respectively. The PCR reaction was carried out using a 25 μl reaction volume with the TopTaq DNA Polymerase kit (Transgen, China). The PCR conditions were as follows: initial denaturation at 94°C for 2 minutes, followed by 30 cycles of denaturation at 94°C for 20 seconds, annealing at 55°C for 30 seconds, and extension at 72°C. A final extension step was performed at 72°C for 10 minutes, followed by storage at 4°C. The amplified products were then subjected to agarose gel electrophoresis. A positive band of 291 base pairs (bp) in length indicated successful target amplification. If no band or only indistinguishable bands were observed, the amplification was considered negative after confirmation by two independent PCR technicians.

mNGS was carried out according to the protocol described previously (Huang et al., 2020b). Briefly, DNA was carefully extracted from the specimens following the kit protocol. The extracted DNA was then thoroughly fragmented into small fragments of approximately 200-300 bp in size and amplified by PCR. Then, the PCR amplicons were subsequently prepared as DNA nanoballs and loaded onto sequencing chips. The sequencing platform used was BGISEQ-500 (BGI Genomics).

### Interpretation of microbiological results

Contamination of the microbiological culture results was considered in the following situations: 1) identification of common skin or hair follicle colonizers that have not been reported in bone and joint infections, and 2) consistent identification of the same microorganism in multiple specimens without corresponding clinical characteristics. To confirm the true positivity of mNGS results in cases where microbiological culture was negative but mNGS was positive, or when microbiological culture indicated a single-pathogen infection while mNGS suggested a mixed infection, the following approaches were employed: Confirmation through a third method, such as 16S PCR, that produced consistent results with mNGS; or optimization of culture based on mNGS results. Consideration of previous reports of the identified microorganism in bone and joint infections that matched the patient’s clinical characteristics. Exclusion of microorganisms known as skin or hair follicle colonizers (e.g., Propionibacteria, Corynebacteria), reagents (e.g., Contaminated by Acinetobacter spp.), laboratory contaminants (e.g., Ralstonia spp., Burkholderia spp.), and other exogenous microorganisms introduced during laboratory procedures.

### Interpretation of microbiological results and treatment outcome evaluation

Microbiological culture and sequencing findings were interpreted by an expert panel consisting of at least one senior microbiologist, one senior infectious disease specialist, and one senior PJI specialist. Treatment success was evaluated based on the 2013 international consensus criteria (Diaz-Ledezma et al., 2013), as detailed in **Supplementary Material 1**.

### Targeted antibiotic therapy and complications

Targeted antibiotic therapy entails isolating causative pathogens through culture and selecting antibiotics based on susceptibility profiling. Antibiotic complications were defined according to previously published criteria (Xu et al., 2022), as detailed in **Supplementary Material 1**.

### Follow up

Demographic information, serum inflammatory markers (including erythrocyte sedimentation rate, ESR; C-reactive protein, CRP; synovial fluid polymorphonuclear granulocyte percentage, SF-PMN; and synovial fluid white blood cell count, SF-WBC), culture, PCR and mNGS results were documented for all patients. The utilization of three diagnostic strategies, pathogen detection rate, targeted antibiotic prescription rate, antibiotic complication rate and infection control rate were concurrently recorded across the three stages. All patients were followed up for a minimum of 2 years.

### Statistical analysis

Statistical analyses were performed using SPSS 21.0 (SPSS Inc., Chicago, IL, USA). Categorical variables were compared among the three groups using chi-square test or Fisher’s exact test. Bonferroni correction was applied to adjust the significance level for multiple comparisons; P values less than 0.017 were considered statistically significant. One-way analysis of variance (ANOVA) was utilized for comparisons among the three groups when data were normally distributed, while independent specimens t-test was used for comparisons between two normally distributed groups. Sensitivity, specificity, positive predictive value (PPV), negative predictive value (NPV), and accuracy were calculated for each diagnostic method.

## Results

### Demographic characteristics

Based on the inclusion criteria, 230 suspected PJI cases were initially identified. Among these, 190 were confirmed as PJI while 11 cases were excluded due to insufficient medical data, 9 due to infections at other sites, and 5 due to suspected specimen contamination. Finally, 165 PJI cases were enrolled, with 28 in Stage A, 62 in Stage B, and 75 in Stage C. No significant differences in age, gender, joint site, preoperative ESR, CRP, SF-WBC or SF-PMN% were found among the three stages (P>0.05). Additionally, 40 cases were diagnosed as AF cases, but 2 were excluded owing to incomplete records. Therefore, Therefore, 38 cases of AF from three stages were included, with 8 cases in Stage A, 13 cases in Stage B, and 17 cases in Stage C. No significant demographic or joint site differences were observed between the PJI and AF cohorts (P>0.05), as shown in **Table 1**.

**Table 1:** Demographic characteristics of PJI and AF in different stages.

Parameters	Total (n=165)	Stage A (n=28)	Stage B (n=62)	Stage C (n=75)	P Value** (Avs. B vs. C)	AF (n=38)	P Value* (Total vs. AF)
Female(n)	94	16	38	40	0.64^a^	24	0.49^a^
Age, yrs (SD)	65.41 ± 9.57	67.54 ± 11.50	64.15 ± 10.03	65.67 ± 8.30	0.29^b^	62.0 ± 12.82	0.09^c^
BMI, kg/m2 (SD)	25.45 ± 3.34	25.54 ± 4.17	25.08 ± 2.81	25.45 ± 3.34	0.55^b^	26.01 ± 2.69	0.34^c^
Joint involved (n)							
Hip	80	13	28	39	0.71^a^	17	0.68^a^
Knee	85	15	34	36		21	
Sinus (n)	35	5	13	17	0.87^a^	0	
Median CRP, mg/L(IQR)	39.12(8.06 to 67.20)	48.15(18.15 to 70.75)	41.02(5.56 to 79.41)	34.18(7.58 to 55.80)	0.15^d^	7.00(5.95 to 9.00)	<0.001^e^
Median ESR, mm/h(IQR)	57.78(33.00 to 79.50)	59.32(34.25 to 89.00)	60.27(29.75 to 85.25)	55.16(33.00 to 76.00)	0.87^d^	17.00(13.75 to 23.00)	<0.001^e^
Median SF-WBC,10^6^/l(IQR)	36269.00(5943.00 to 46347.50)	40607.00(7801.25 to 42676.75)	37567.00(3717.50 to 34541.50)	33575.00(6040.00 to 54780.00)	0.18^d^	832.00(667.00 to 966.00)	<0.001^e^
Median SF-PMN%(IQR)	84.70(78.70 to 90.45)	85.10(81.65 to 91.03)	83.40(78.57 to 90.13)	82.70(78.40 to 90.30)	0.27^d^	49.00(43.75 to 55.25)	<0.001^e^

a Chi-squared test.

b One-way ANOVA test.

c Independent-samples t-test.

d Kruskal-Wallis H test.

e Mann-Whitney U test.

PJI, periprosthetic joint infection; Total, Stage A+B+C; AF, aseptic failure; ESR, erythrocyte sedimentation rate; CRP, C-reactive protein; SF-WBC, synovial fluid white blood cell; SF-PMN%, synovial fluid polymorphonuclear neutrophil percentage.

*P<0.05, **P<0.017.

### Utilization rates of different optimization protocols across three stages

**Figure 1:**
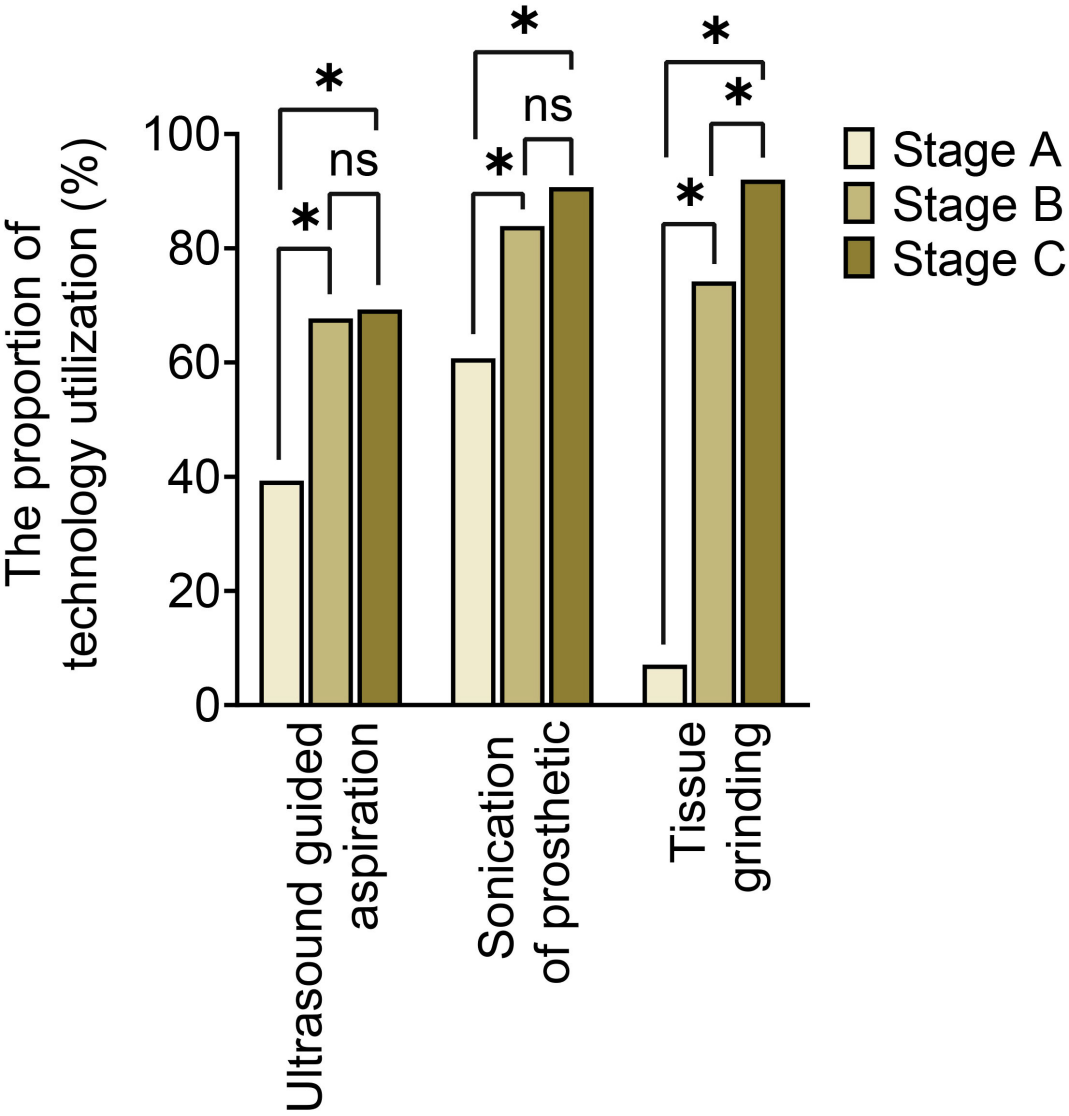
Sample collection optimization across different stages. *:P<0.017, ns, No Significance.

**Figure 2:**
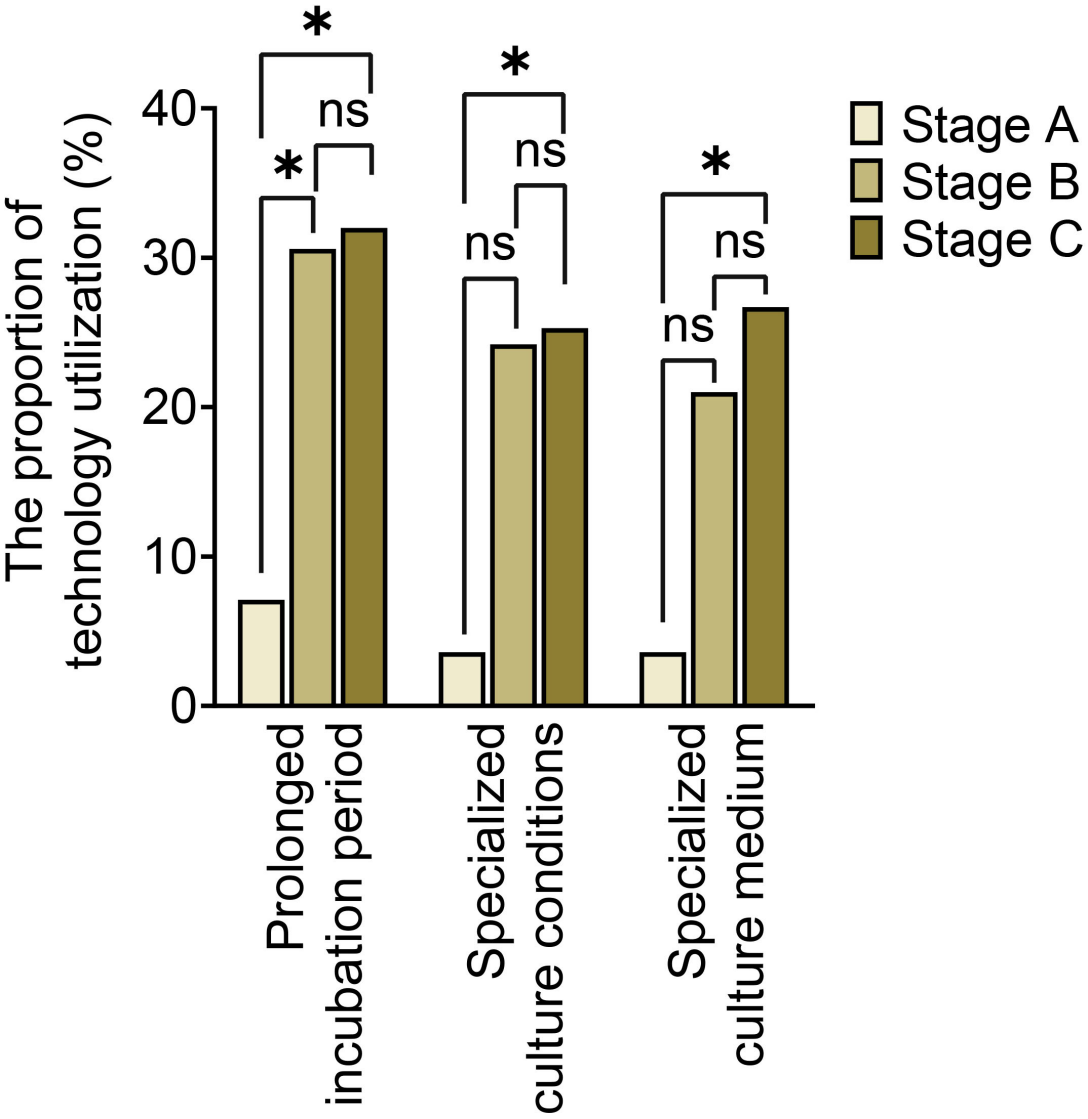
Culture conditions optimization across different stages. *:P<0.017, ns, No Significance.

**Figure 3:**
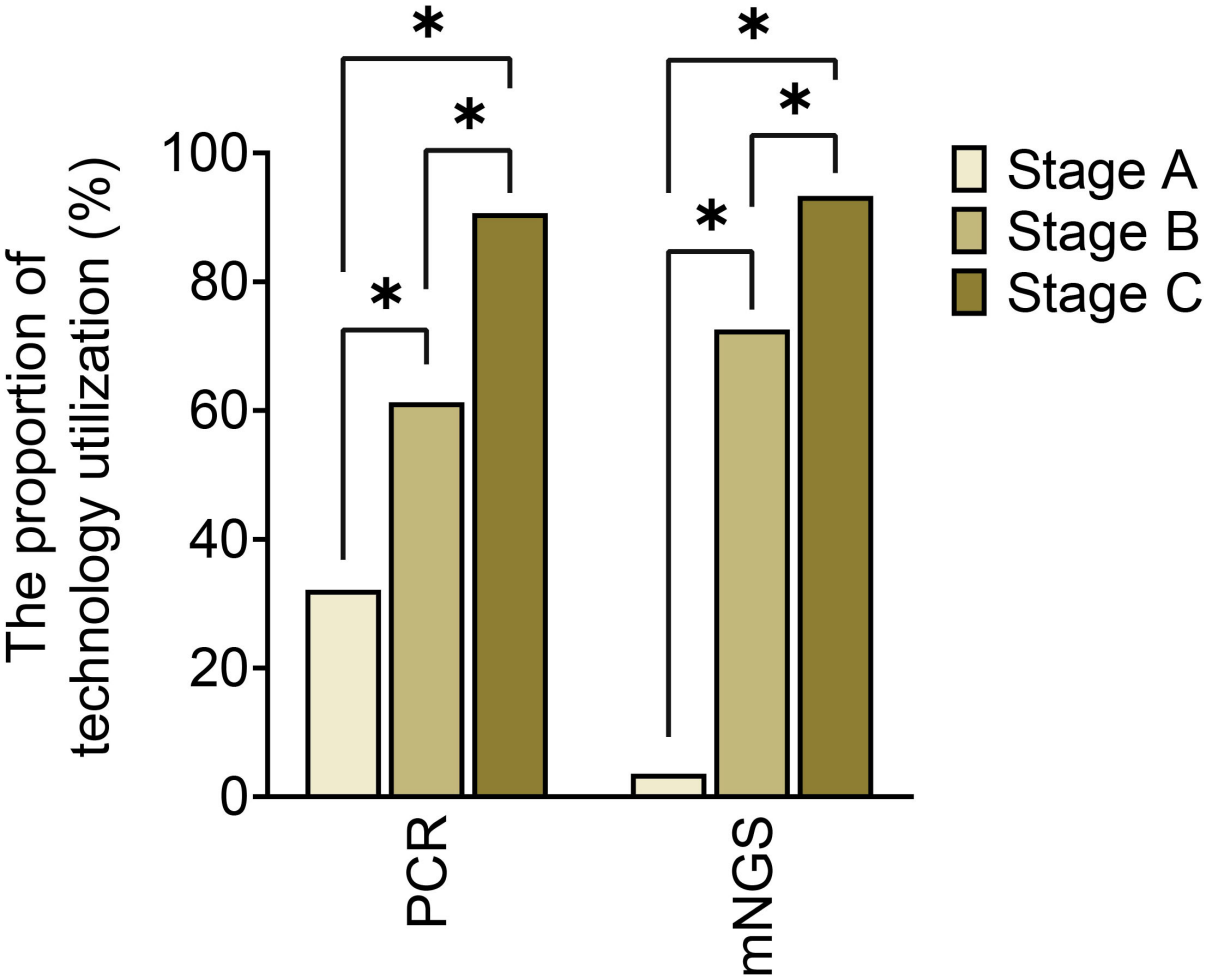
Molecular diagnosis across different stages. *:P<0.017, ns, No Significance.

The utilization of optimized specimen acquisition techniques exhibited a gradual increase across the three stages. During stages B and C, the usage rates of ultrasound-guided puncture and sonication of explanted prostheses were significantly higher compared to stage A (P<0.017; P<0.017) (as shown in **Figure 1**). Furthermore, significant differences were found in all pairwise comparisons of tissue grinding utilization among stages A, B, and C (P<0.017; P<0.017;P<0.017). Regarding optimized cultivation techniques (as shown in **Figure 2**), no significant differences were observed between stages B and C in terms of prolonged incubation periods, specialized culture conditions, or customized media (P>0.017; P>0.017). However, utilization rates of optimized cultivation in stage C were markedly higher than in stage A (P<0.017;P<0.017). For molecular diagnostics utilization (as shown in **Figure 3**), PCR and mNGS usage rates in stage C were significantly elevated compared to stages A and B (P<0.017;P<0.017).

### Comparison of pathogen detection rate, targeted antibiotic rate, and infection control rate across three stages

**Figure 4:**
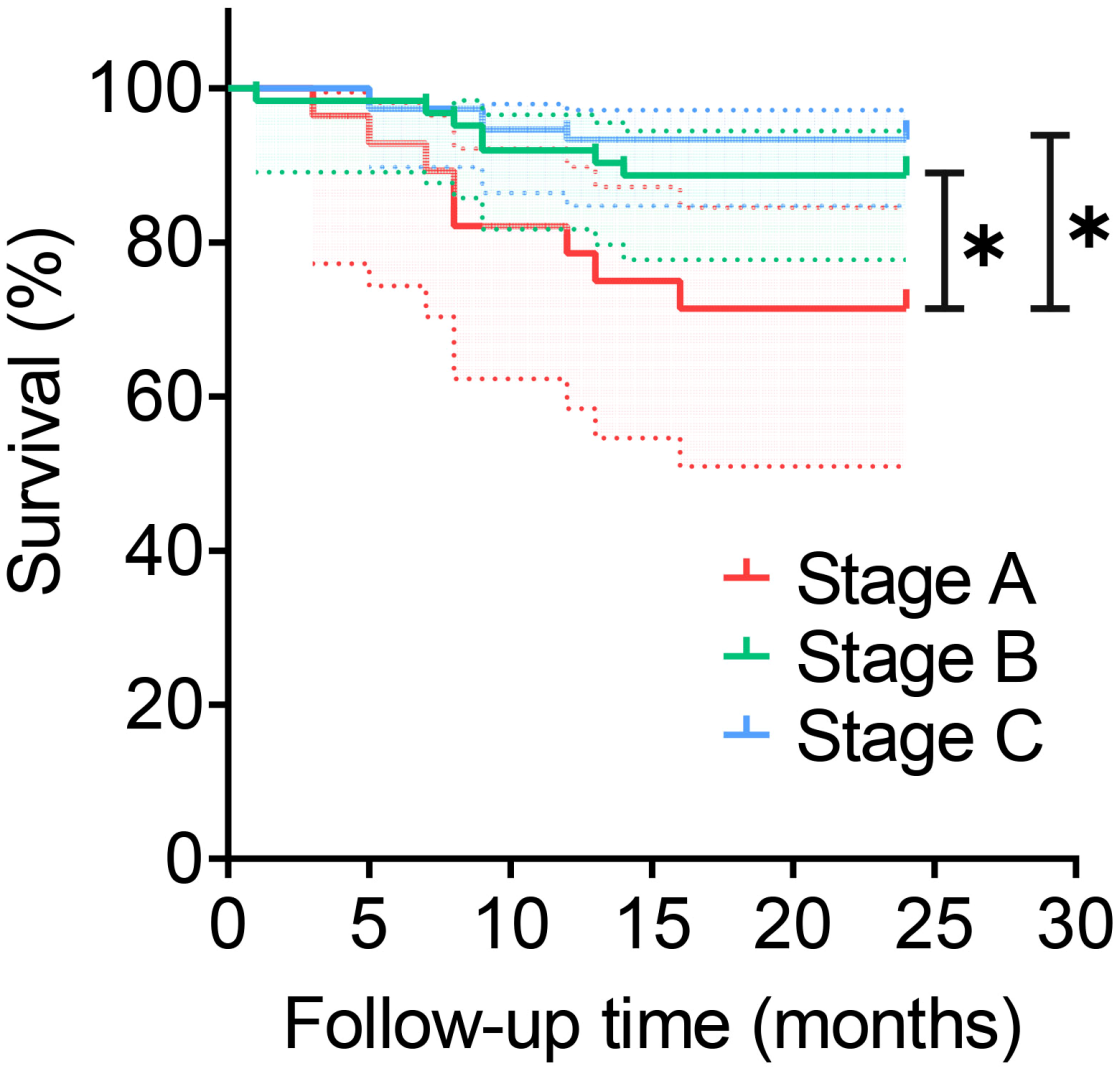
Survival rate of different stages. *:P<0.017. This dotted line represents the 95% confidence interval.

The stages B and C demonstrated significantly higher pathogen detection rates, targeted antibiotic prescription rates, and infection control rates compared to stage A, as shown in **Table 2**. However, stage A exhibited a markedly higher incidence of antibiotic-related complications compared to stages B and C. No significant differences among the three stages regarding polymicrobial PJI detection rates. In terms of infection control, the infection control rates in stages B (90.3%,56/62) and C (93.3%,70/75) were both above 90% during the 2-year follow-up after surgery. The difference was not statistically significant (P > 0.017), but both were higher than that of group A (71.4%,20/28), which showed a statistically significant difference (P<0.017, P<0.017), as shown in **Table 2**. And the Kaplan-Meier survival curve in **Figure 4**. Additionally, the combination of all three optimization protocols yielded the highest sensitivity of 91.21% for PJI detection, while retaining higher specificity of 92.11%, as shown in **Table 3**.

**Table 2:** Comparison of pathogen detection rate, antibiotic targeting rate, antibiotic complication rate, and infection control rate in three stages.

Variable, n(%)	Stage A (n=28)	Stage B (n=62)	Stage C (n=75)	χ2 Value	P Value
Pathogen detection rate	19(67.9%)^*#^	53(85.5%)	68(90.7%)	8.282	0.016^a^
Antibiotic targeting rate	16(57.1%)^*#^	51(82.3%)	63(84.0%)	9.515	0.009^a^
Antibiotic complication rate	9(32.1%)^*#^	7(11.3%)	9(12.0%)	7.586	0.023^a^
Infection control rate	20(71.4%)^*#^	56(90.3%)	70(93.3%)	8.317	0.011^a^
Polymicrobial Infection	2(7.1%)	8(12.9%)	13(17.3%)	1.641	0.479^b^

*Compared to Stage B, have a statistically significant difference.

#Compared to Stage C, have a statistically significant difference.

a Bonferroni test.

b Chi-square test.

**Table 3:** Analysis of sensitivity and specificity of different methods and their combinations for diagnosing PJI.

Variable (PJI)	PJI (Pathogen positive)	Non-PJI (n=38, Pathogen positive)	Sensitivity	Specificity	Youden’s index	PPV	NPV	LR+	LR-
without any optimization (n=46)	24	1	52.17%	97.37%	0.50	96.00%	62.71%	19.83	0.50
Optimizing Specimen Acquisition (n=119)	77	1	64.71%	97.37%	0.62	98.72%	46.84%	24.59	0.36
Optimizing Culture Conditions (n=106)	80	2	75.47%	94.74%	0.70	97.56%	58.06%	14.34	0.26
Molecular Diagnostics (n=127)	108	3	85.04%	92.11%	0.77	97.30%	64.81%	10.77	0.16
Optimizing Specimen Acquisition + Optimizing Culture Conditions (n=99)	79	3	79.80%	90.63%	0.70	96.3%	59.2%	8.51	0.22
Optimizing Culture Conditions + Molecular Diagnostics (n=93)	80	3	86.02%	92.11%	0.78	96.39%	72.92%	10.90	0.15
Optimizing Specimen Acquisition + Molecular Diagnostics (n=108)	93	3	86.11%	92.11%	0.78	96.88%	70.00%	10.91	0.15
Combining Three Optimization Approaches (n=91)	83	3	91.21%	92.11%	0.83	96.51%	81.40%	11.55	0.10

Youden’ s index = sensitivity + specificity – 1; Combining Three Optimization Approaches, Optimizing Specimen Acquisition + Optimizing Culture Conditions + Molecular Diagnostics.

### Optimal strategy for pathogen diagnostic workflow

**Figure 5:**
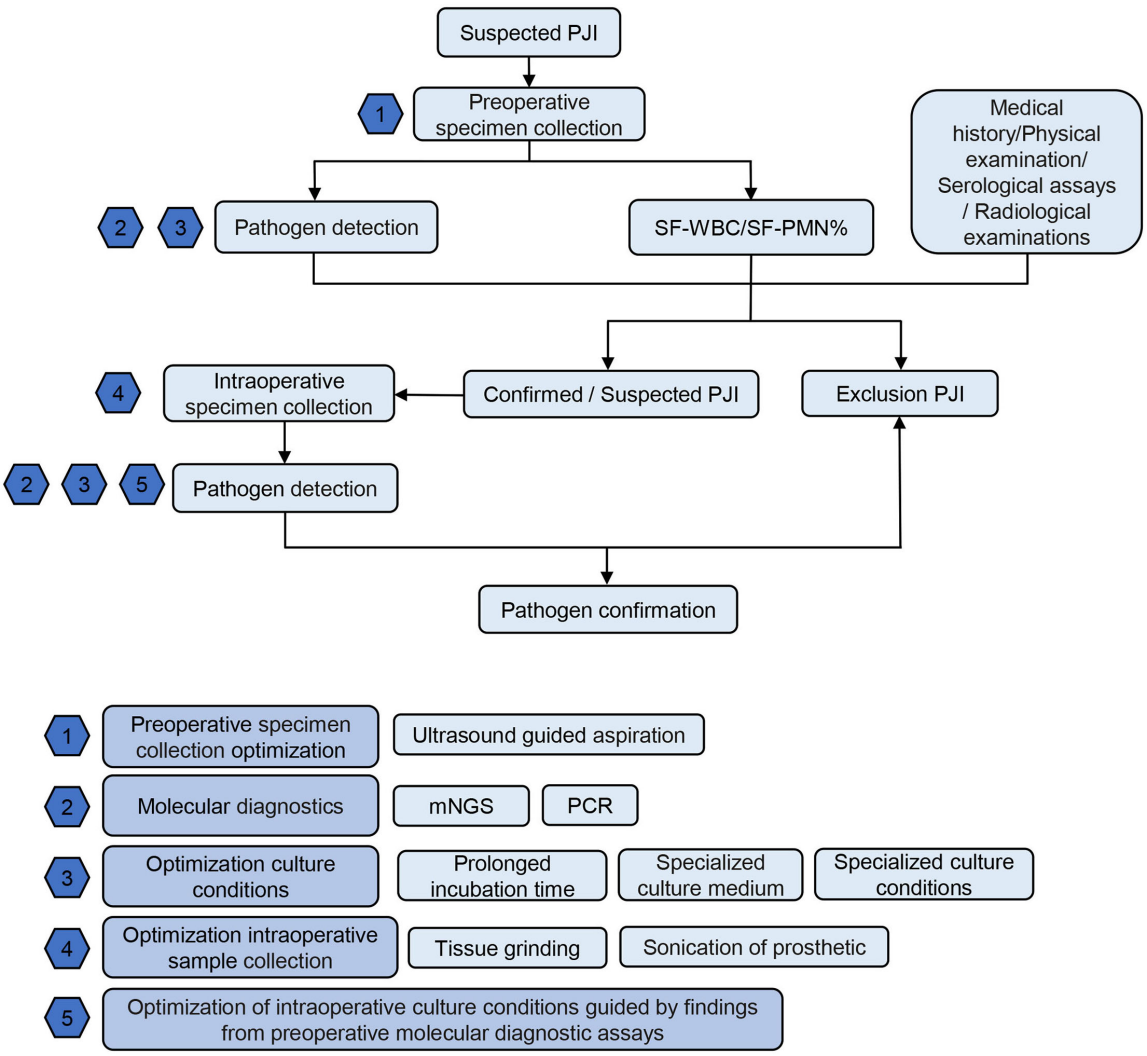
Recommended PJI Pathogen Detection Flowchart. PJI, periprosthetic joint infection; SF-WBC, synovial fluid white blood cell count; SF-PMN%, synovial fluid polymorphonuclear granulocyte percentage; mNGS: metagenomic next-generation sequencing; PCR polymerase chain reaction.

**Figure 5** depicts the workflow for PJI pathogen detection in our center. In brief, for suspected PJI patients, preoperatively, joint aspiration and synovial fluid sampling are performed under ultrasound guidance. The synovial fluid undergoes white blood cell count/classification and pathogen detection, including molecular diagnostics and optimization of culture conditions. This is complemented by medical history, physical examination, serological tests, and imaging studies. If the results are negative, PJI is ruled out. If PJI is confirmed or suspected, intraoperative specimen collection is optimized for pathogen detection. For specific PJI cases, such as suspected Gram-negative or anaerobic bacterial PJI, appropriate culture conditions are selected. In cases of PJI with sinus tract formation, deep tissue grinding culture is recommended. For explanted PJI prostheses or components, sonication prosthesis disruption fluid is collected for testing.

### Special cases

Case 1 presents a chronic PJI with a history of antibiotic use. Preoperative synovial fluid aspiration culture yielded negative results, while metagenomic mNGS indicated the presence of Staphylococcus aureus (SA). Intraoperative sonication prostheses fluid and tissue cultures were also negative. Considering the patient’s history of antibiotic use and the intracellular persistence characteristics of SA, tissue grinding culture was performed, eventually resulting in the isolation of SA. Case 2 presents a chronic PJI in which conventional culture methods yielded negative results both preoperatively and intraoperatively. However, preoperative synovial fluid mNGS indicated the presence of Mycoplasma hominis. Intraoperatively, sonication fluid and tissue were cultured using liquid media and a selective solid culture medium for Mycoplasma (provided by Haibio Technology). Ultimately, Mycoplasma hominis was isolated. Case 3 is a chronic PJI without evident joint redness, swelling, or pain and no history of antibiotic use. Preoperative synovial fluid culture yielded negative results, while mNGS suggested the possible presence of Mycobacterium avium. We optimized the culture method used in Case 4, and eventually, Mycobacterium avium was isolated from the intraoperative sonication fluid and tissue. Case 4 involves a chronic PJI with a history of antibiotic use. Preoperative synovial fluid aspiration, intraoperative tissue, and sonication fluid cultures, as well as PCR testing, all yielded negative results. However, mNGS indicated the presence of Candida parapsilosis. Case 5 is a PJI with sinus tract formation. Preoperative synovial fluid culture and mNGS detected Staphylococcus epidermidis and Escherichia coli. Intraoperatively, both tissue grinding and sonication prostheses fluid cultures confirmed the presence of Escherichia coli. Verification through 16S rDNA PCR and subsequent culture only confirmed the presence of Escherichia coli, suggesting that Staphylococcus epidermidis was a contaminant. Case 6 involves an acute hematogenous PJI following tooth extraction, manifested by acute fever and joint redness and swelling. Preoperatively, elevated levels of ESR, CRP, SF-WBC, and SF-PMN% were observed. Synovial fluid culture was negative. Preoperative synovial fluid mNGS revealed Aggregatibacter aphrophilus, which was subsequently isolated on blood agar plates using prolonged anaerobic incubation in 5% CO2 for 12 days.

## Discussion

Identification of causative pathogens is crucial for the successful management of PJI (Tzeng et al., 2015). Various enhanced techniques have been proposed to improve detection rates, including sonication of explanted prostheses, prolonged culture duration, specialized media, and mNGS (Trampuz et al., 2007; Zhang et al., 2019; Cai et al., 2020; Fang et al., 2021b). Our institution has implemented nearly all reported optimization methods for PJI pathogen detection, achieving over 90% detection rate. With optimization of molecular diagnostics, culture conditions, and intraoperative specimen collection, we observed increased pathogen detection and targeted antibiotic utilization. However, when utilizing a combined diagnostic approach, interpretation of results may become more complex. Thus, when utilizing combined methods, integration of patient history, symptoms, examination findings, laboratory tests, and specialist consultation is recommended to enable targeted antibiotic therapy and reduce antibiotic complications. This collaborative approach aims to achieve optimal PJI treatment outcomes.

We recommend applying optimized pathogen detection strategies based on the characteristics of individual PJI cases. For prosthesis removal surgery, sonication fluid from the removed hardware should be obtained,given the propensity of pathogens to form biofilms on implant surfaces. Zhang et al. demonstrated higher detection rates with sonication fluid culture and mNGS compared to conventional culture (Zhang et al., 2019). Importantly, to avoid contamination, the prosthesis should be placed directly in a sterile, sealed container during the surgical procedure, rather than in a plastic bag. In cases where preoperative joint aspiration is not feasible, tissue homogenization is advised to facilitate pathogen release and microbial load. Fang et al. showed improved culture positivity with tissue grinding (Fang et al., 2021b). For suspected intracellular PJI pathogens such as Listeria monocytogenes (Henry et al., 2010), Mycobacterium tuberculosis (Mayer-Barber et al., 2014), and Legionella pneumophila (Naujoks et al., 2016), tissue grinding is recommended prior to processing. Extended 2-week incubation periods are advisable when culturing fastidious organisms such as Propionibacterium acnes and coagulase-negative staphylococci (Zimmerli et al., 2004; Zappe et al., 2008).

For culture-negative but mNGS-positive cases of periprosthetic joint infection (PJI), tailoring specialized media and incubation based on mNGS results can enhance pathogen identification (Fang et al., 2021a). Antibiotic use before sampling often leads to negative cultures by inhibiting microorganism growth, a significant challenge alongside the limitations of traditional cultures to grow dormant or biofilm-encased organisms. Up to 10% of conventional cultures fail to isolate pathogens, including those needing extended incubation like Propionibacterium acnes (Zimmerli et al., 2004; Bejon et al., 2010). Biofilms further complicate detection, as standard methods focus on planktonic bacteria (Kalbian et al., 2020). Addressing these issues, employing intraoperative techniques like tissue grinding and sonication, along with molecular diagnostics (PCR, mNGS), promises improved detection rates and outcomes in PJI management.

In suspected false-positive mNGS cases, broad-range or multiplex PCR and optimized culture techniques are suggested for reconfirmation. In a prior institutional study (Fang et al., 2021a), optimized intraoperative sampling based on preoperative mNGS increased sensitivity, specificity, and accuracy to 94.29%, 76.19%, and 87.5%, versus 60%, 80.95% and 67.86% with conventional culture, respectively, for 35 prosthetic joint infections and 21 non-infectious cases.

In PJI cases with sinus tract formation, culturing sinus secretion is not advised due to contamination risks (Lluís Font-Vizcarra et al., 2010). Instead, tissue grinding culture and mNGS of intraoperative specimens from the prosthetic vicinity are recommended. If contamination is suspected in detection results, PCR can be utilized for further validation.

In the management of PJI, the incorporation of optimized pathogen detection strategies incurs additional costs. However, considering the potential for improved clinical outcomes and long-term cost savings, such strategies merit careful consideration. Conducting a comprehensive cost-benefit analysis tailored to the specific circumstances of each healthcare setting is crucial for making informed decisions about adopting these advanced diagnostic technologies. This study had some limitations:1). The sample size included was relatively small, potentially resulting in insufficient statistical power for analysis; 2). As a single-center retrospective study based solely on data from one medical institution, the generalizability of the research conclusions may be limited to some extent; 3). Due to the short follow-up duration, this study lacked long-term follow-up data, precluding the assessment of treatment durability. However, to the best of our knowledge, this is the first study to analyze and compare utilization of different diagnostic methods across three stages, evaluate their clinical effectiveness, and propose an optimal workflow for PJI pathogen detection.

## Conclusion

In summary, this study preliminarily demonstrates that the integrated application of multiple detection strategies, including optimized sampling, optimized culture conditions, and molecular diagnostics, can significantly improve the detection rate of causative pathogens in PJI. This approach enables the formulation of more precise targeted antibiotic treatment regimens, effectively reducing antibiotic-related complications and ultimately improving the clinical outcomes for PJI patients.

## Data availability statement

The data presented in the study are deposited in the China National GeneBank Database (CNGBdb) repository, accession number CNP0001047 (http://db.cngb.org/cnsa/).

## Ethics statement

The studies involving humans were approved by First Hospital of Fujian Medical University. The studies were conducted in accordance with the local legislation and institutional requirements. The participants provided their written informed consent to participate in this study. Written informed consent was obtained from the individual(s) for the publication of any potentially identifiable images or data included in this article.

## Author contributions

QW: Data curation, Formal analysis, Software, Writing – original draft. YFC: Formal Analysis, Writing – original draft, Conceptualization. YC: Writing – original draft, Resources, Validation. JL: Validation, Writing – original draft, Methodology, Visualization. HD: Visualization, Writing – original draft, Data curation. JGH: Visualization, Writing – original draft, Investigation. JXH: Writing – original draft, Formal Analysis, Validation. ZH: Writing – original draft, Data curation, Resources. BY: Writing – original draft, Methodology, Visualization. WZ: Funding acquisition, Supervision, Writing – review & editing. XF: Funding acquisition, Writing – review & editing, Project administration.
