# Supplementary material for: Improved cure rate of periprosthetic joint infection through targeted antibiotic therapy based on integrated pathogen diagnosis strategy
Source: Front Cell Infect Microbiol. 2024 May 21;14:1388385. doi: 10.3389/fcimb.2024.1388385 (PMC11148460; doi:10.3389/fcimb.2024.1388385)
Supplement: Supplementary file 1 [file DataSheet_1.doc]

**Supplementary Material 1**

**Methods**

**Interpretation of Microbiological Results**

Contamination of the microbiological culture results was considered in the following situations: 1) identification of common skin or hair follicle colonizers that have not been reported in bone and joint infections, and 2) consistent identification of the same microorganism in multiple samples without corresponding clinical characteristics. To confirm the true positivity of mNGS results in cases where microbiological culture was negative but mNGS was positive, or when microbiological culture indicated a single-pathogen infection while mNGS suggested a mixed infection, the following approaches were employed: Confirmation through a third method, such as 16S PCR, that produced consistent results with mNGS, or optimization of culture based on mNGS results. Previous reports of the identified microorganism in bone and joint infections that matched the patient's clinical characteristics. Exclusion of microorganisms known as skin or hair follicle colonizers (e.g., propionibacteria, diphtheroids), reagents (e.g., Acinetobacter), laboratory contaminants (e.g., Ralstonia spp., Burkholderia spp.), and other exogenous microorganisms introduced during laboratory procedures.

**Evaluation of Treatment Outcome**

which included three aspects: (i) infection eradication, characterized by wound healing without discharge, sinus tract, or wound pain; (ii) absence of infection-related surgical interventions after re-implantation; and (iii) no infection-related deaths (caused by sepsis, necrotizing fasciitis, etc.).

**Complications of antibiotics**

Specifically, antibiotic-related complications include:(i) Bone marrow suppression: Preoperative routine white blood cell count greater than 4 × 10^9/L, with the highest white blood cell count during intravenous or oral antibiotic use falling below 3 × 10^9/L.(ii) Hepatic dysfunction: Preoperative levels of alanine aminotransferase (ALT) and aspartate aminotransferase (AST) within the normal range, with peak values during intravenous or oral antibiotic use increasing by more than 1.5 times. (iii) Renal dysfunction: Complications indicating renal impairment are characterized by preoperative markers of normal renal function, specifically creatinine levels within the normal range, with an increase in creatinine values exceeding 1.5 times the initial value during intravenous or oral antibiotic use.

The data presented in the study are deposited in the China National GeneBank Database (CNGBdb) repository, accession number CNP0001047 (http://db.cngb.org/cnsa/).
